# Functional Analysis of the GH16 Domain-Containing *XTH2* Homologs in Mediating Sunflower Response to *Orobanche cumana* Parasitism

**DOI:** 10.3390/plants15142222

**Published:** 2026-07-21

**Authors:** Yannan Li, Ruonan Yu, Rui Xu, Hada Wuriyanghan, Fang Yan

**Affiliations:** 1 School of Life Science, Inner Mongolia University, Hohhot 010030, China; lyn15848497757@163.com (Y.L.); 15071201086@163.com (R.X.); nmhadawu77@imu.edu.cn (H.W.); 2 Key Laboratory of Herbage & Endemic Crop Biology, Ministry of Education (IMU), Hohhot 010030, China; 3 College of Agriculture, Hulunbuir University, Hulunbuir 02100, China; yuruonan2016@sina.com

**Keywords:** *Helianthus annuus*, *Orobanche cumana*, XTH2 homologs, GH16 domain, cell wall remodeling

## Abstract

Sunflower (*Helianthus annuus*) is highly susceptible to infection by the root parasitic plant *Orobanche cumana* during its growth. In establishing connections with the sunflower root system, *O. cumana* induces the hydrolysis and remodeling of the host cell wall. Xyloglucan endotransglucosylase/hydrolase (XTH), a member of glycoside hydrolase family 16 (GH16), is a key enzyme involved in the hydrolysis and synthesis of xyloglucan, playing a critical role in cell wall modification and reconstruction. However, the involvement of *XTH* families in the interaction between sunflower and *O. cumana* remains unclear. In this study, we showed that the expression level of *HaXTH2* was upregulated in sunflowers following *O. cumana* infection. Overexpression of *HaXTH2* loosens host primary cell walls and represses lignin-based defense responses at the early infection stage, thereby facilitating haustorial penetration across cortical tissues and xylem bridge formation to support normal parasitism of *O. cumana*. Functional analysis revealed that *HaXTH2* and its homolog, *HaXTH2-1*, facilitate *O. cumana* infection, whereas *OcXTH2*, an *O. cumana* homolog, suppresses this process. Furthermore, HaXTH2, HaXTH2-1, and OcXTH2 are localized to the cell wall. Domain truncation analysis revealed that the GH16 domain alone from HaXTH2 and HaXTH2-1 enhance parasitic susceptibility, while the xyloglucan endotransglycosylase C-terminal domain (XET_C) domain does not. Mutation analysis identified Y20 in *HaXTH2* and H96 in *HaXTH2-1* as key amino acid sites regulating *O. cumana* parasitism. This study expands our understanding of the functions of *XTHs* in plant–plant interactions and provides a theoretical basis for further development of *O. cumana*-resistant sunflower cultivars.

## 1. Introduction

Sunflower (*Helianthus annuus* L.), an *Asteraceae* species originating from North America, is one of the most economically important oilseed crops worldwide. Sunflower yield is constrained by a range of biotic and abiotic stresses, including drought, frost, waterlogging, pathogens, insect pests, and parasitic plants. The interaction between sunflower and parasitic plants represents an especially sophisticated biological process [1,2]. Under stress conditions, sunflowers activate multiple adaptive responses, such as delayed leaf senescence, upregulated expression of photosynthesis-related genes, improved nutrient accumulation, and altered cell wall structure [3,4,5].

Most plants are autotrophs, but approximately 4750 angiosperm species are parasitic, relying wholly or partly on host plants to extract water and nutrients for survival [2]. Based on the parasitism style, parasitic plants can be classified into root parasitic plants and stem parasitic plants. Sunflower broomrape (*Orobanche cumana* Wallr.) is a destructive root parasite that predominantly infects cultivated sunflower. Parasitic colonization on sunflower roots severely impairs crop yield and quality, resulting in annual economic losses exceeding ten billion US dollars in Asia and Africa [6]. It is a substantial threat in Europe, especially in countries around the Black Sea and in Spain. *O. cumana* has a negative effect on sunflower development. The infected plants are smaller, the sunflower head diameter is reduced, and up to 80% of yield losses are observed [7]. The initiation of parasitism commences with the germination of *O. cumana* seeds, a process induced by germination stimulants exuded from host root systems. The primary germination stimulants secreted by sunflower roots include strigolactones and sesquiterpene lactones. The second stage involves the attachment of *O. cumana* to host roots. Following seed germination, the parasite develops haustoria, which further establishes vascular connections and xylem bridges to anchor itself firmly to sunflower roots. In the third stage, underground tubercles rapidly develop as nutrient storage organs [8,9]. During this parasitic process, dramatic hydrolysis and remodeling occur in sunflower root cell walls. During the parasitic attachment stage, the developing haustorium penetrates host root tissues and differentiates specialized xylem bridge cells at the host–parasite interface. These unique vascular cells create continuous xylem conduits linking the parasite and sunflower root stele, enabling long-distance transport of water, mineral nutrients and photosynthates. The quantity and integrity of xylem bridges directly determine parasitic colonization success; severe host cell wall lignification will block xylem bridge formation and restrict nutrient delivery to the parasite.

The plant cell wall serves as the primary defense barrier for perceiving “danger signals” and forms a critical interface between the plant cell and the external environment [10]. When exposed to biotic stresses—such as those inflicted by viruses, bacteria, fungi, nematodes, or insects—the cell wall initiates immediate physical defense responses, including callose deposition, enhanced lignification and suberization, and the cross-linking of cell wall proteins [11,12]. Cell wall-modifying enzymes are essential for reinforcing these defense mechanisms. The cell wall is primarily composed of three layers: the primary cell wall, the secondary cell wall, and the middle lamella [13]. These layers are predominantly constructed from polysaccharides, including cellulose, hemicellulose, lignin, and pectin, providing structural support, protection, and facilitating cell proliferation and expansion [14].

Cell proliferation and volume expansion of plant cells rely heavily on cell wall remodeling [15]. Xyloglucan (XyG) is a major hemicellulose constituent of the primary cell wall in higher plants. It features a cellulose-like backbone decorated with oligosaccharide side chains, each consisting of multiple xylose residues [16]. Dynamic cell wall remodeling depends on the cleavage and re-synthesis of xyloglucan [17]. XTH mediates the cleavage and re-ligation of xyloglucan chains and remodels the cellulose–xyloglucan network, serving as a core regulator of cell wall dynamics [18]. XTHs were among the first enzymes shown to exhibit catalytic activity in vitro. XTH family proteins typically contain two conserved domains: the Glyco_hydro_16 domain (glycoside hydrolase family 16) and the XET_C domain (xyloglucan endotransglycosylase C-terminal domain). These domains are responsible for the enzymatic activity of XTH proteins in modifying cell wall xyloglucans. Biochemical assays have validated that XTH proteins possess both xyloglucan endotransglucosylase (XET) and xyloglucan endohydrolase (XEH) activities [19], which are indispensable for cell wall restructuring [20,21]. During plant development, XTH proteins contribute to cell wall loosening, synthesis, strengthening, and degradation [22]. To date, the *XTH* gene family has been widely identified across plant species: 33 members in *Arabidopsis thaliana* [23], 29 in *Oryza sativa* [24], 41 in *Populus* L. [25], and 25 in *Solanum lycopersicum* [26]. Homologs have also been reported in *Nicotiana tabacum* L., *Glycine max*, *Hordeum vulgare* L., and *Ananas comosus*.

Functional studies in *Arabidopsis thaliana* have demonstrated that *AtXTH17* and *AtXTH18* are specifically expressed in root meristematic and elongation zones. *AtXTH1*8 is essential for constructing and remodeling the xyloglucan-cellulose network during cell elongation and root maturation [27,28]. Mutation of *AtXTH27* disrupts vascular tissue development in rosette leaves [29]. *AtXTH28* regulates stamen elongation, and its dysfunction leads to decreased self-pollination efficiency [30]. Silencing of *AtXTH31* and *AtXTH32* strongly suppresses cell wall expansion, highlighting the essential function of *XTH* genes in cell wall assembly and disassembly [31,32]. In tomato, *LeXTH1* facilitates hypocotyl wall loosening and cell elongation during seed germination [33]. Rice *OsXTH8* is induced by gibberellin (GA_3_) and plays a critical role in shoot growth [34]. *DkXTH1* modulates fruit ripening and early expansion [35]. Previous research on the tomato-*Cuscuta chinensis* parasitic system showed that host *LeXTH1* enhances cell wall compaction to resist parasitic infection [36,37]. Conversely, *C. chinensis* exhibits elevated expression of *XTH* genes in haustoria to loosen host cell walls and accelerate invasion. This opposite functional pattern reveals the diverse roles of *XTH* genes in plant interspecific interactions.

However, host–parasite interactions involve continuous cell wall remodeling, yet the regulatory roles of host and parasitic XTH2 during *O. cumana* infection remain poorly understood. Therefore, this study aimed to characterize the functional divergence between *HaXTH2*/*HaXTH2-1* and *OcXTH2*, and to reveal how their key amino acid residues modulate parasitism.

## 2. Results

### 2.1. Sunflower HaXTH2 Localizes to the Cell Wall and Promotes O. cumana Parasitism

**Figure 1:**
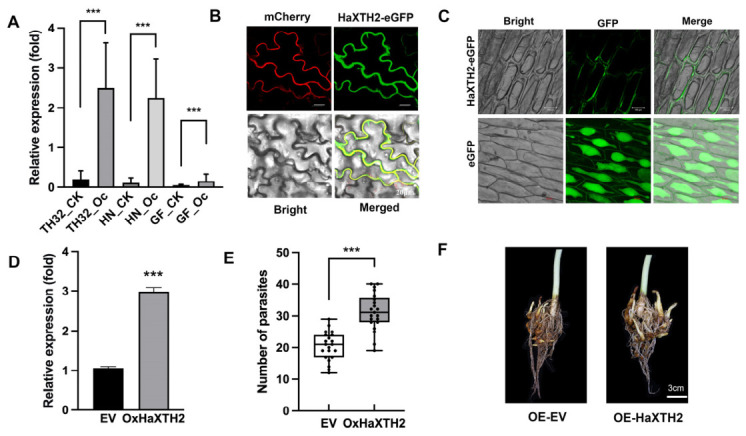
HaXTH2 localizes to the cell wall and promotes *O. cumana* parasitism in sunflowers. (**A**) Expression analysis of *HaXTH2* in the roots of three sunflower cultivars before and after *O. cumana* parasitism. HN, HN3638; GF, GF3638C; CK, non-inoculated control group; Oc, *O. cumana*-inoculated group. Data are means ± SD of three biological replicates (*n* = 3). (**B**) Subcellular localization of the *HaXTH2*-GFP fusion protein in *Nicotiana benthamiana* leaves. *HaXTH2*-GFP represents overexpressed *HaXTH2* fused with GFP, and mCherry was used as a plasma membrane marker. Scale bar = 20 μm. Data are representative of three independent experiments. (**C**) Subcellular localization analysis of *HaXTH2* using onion epidermal cells. *HaXTH2*-GFP served as the experimental group, and GFP served as the control group. From left to right: bright field, green fluorescence, merge. Data are representative of three independent experiments. (**D**) Transcript levels of *HaXTH2* in sunflower roots following transient overexpression, compared with the empty vector control (OE-EV) at 45 days post-inoculation (dpi) with *O. cumana*. Data are means ± SD of three biological replicates (*n* = 3). (**E**) Quantification of *O. cumana* tubercles on roots of OE-*HaXTH2* plants at 45 dpi. Data are means ± SD from three independent experiments, each with 18 biological replicates per group. Asterisks indicate statistically significant differences between experimental and control groups. (**F**) Representative phenotypes of *O. cumana* parasitism on roots of OE-*HaXTH2* plants at 45 dpi. OE-EV represents the empty vector control. Scale bar = 3 cm. Asterisks indicate statistically significant differences between experimental and control groups (one-way ANOVA with Dunnett’s post-hoc test; *** p < 0.001).

This study aimed to identify *XTH* family genes involved in the sunflower–*Orobanche cumana* interaction. The *HaXTH2* gene was first identified from our previous transcriptome data, which were generated from three sunflower cultivars with different resistance levels under inoculated conditions with *O. cumana*. We first examined the expression pattern of *HaXTH2* upon *O. cumana* infection. The results showed that the transcript levels of *HaXTH2* were significantly upregulated after *O. cumana* infection in all three sunflower cultivars (TH32, HN3638, and GF3638C) (Figure 1A). HaXTH2-GFP green fluorescence signals overlapped partially with the red fluorescence of the plasma membrane marker mCherry along the cell periphery (Figure 1B), suggesting that HaXTH2 is present at the peripheral region. However, due to the tight apposition of the cell wall and the plasma membrane, co-localization with a membrane marker alone does not distinguish between these two compartments. To resolve this, plasmolysis experiments were performed (Figure 1C). We employed onion (*Allium cepa*) epidermal cells as an experimental system. Following plasmolysis induced by sucrose treatment, the protoplasts began to detach from the cell wall at the corners of the cells and to contract gradually. Notably, after plasmolysis, the HaXTH2-GFP fluorescence signal did not migrate with the shrinking protoplast, but instead remained associated with the cell wall contour that had separated from the protoplast (Figure 1C), demonstrating that HaXTH2 localizes to the cell wall. We then characterized the role of HaXTH2 in the sunflower during *O. cumana* infection. Transient overexpression of *HaXTH2* in sunflower roots resulted in a 2.9-fold increase in *HaXTH2* transcript levels (Figure 1D). Statistical analysis of *O. cumana* tubercle numbers revealed that *HaXTH2* overexpression (OE) significantly enhanced parasitic numbers compared to the empty vector control (OE-EV) (Figure 1E,F).

### 2.2. HaXTH2 Suppresses Host Cell Wall Lignification to Facilitate O. cumana Parasitism Without Affecting Seed Germination

The entire parasitic lifecycle of *O. cumana* on sunflower occurs underground, making it difficult to directly identify the exact developmental stage regulated by *HaXTH2*. To resolve this issue, we established a *Rhizobium rhizogenes*-mediated sunflower hairy root hydroponic transformation system coupled with an *O. cumana* inoculation assay to monitor early parasitic phenotypes.

**Figure 2:**
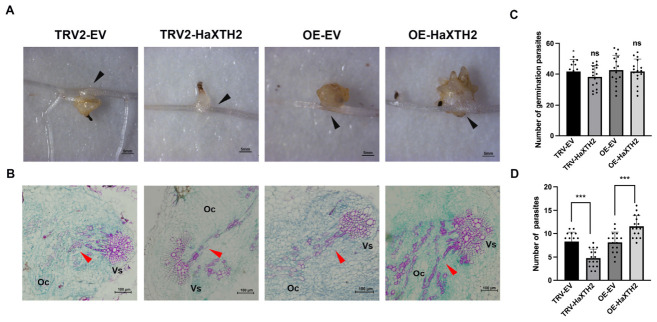
Effects of HaXTH2 on sunflower root development and early parasitism of *O. cumana* in a hydroponic parasitic system. (**A**) Phenotypes of early *O. cumana* parasitism at 15 days post-inoculation (dpi) in the hydroponic system. TRV-EV, VIGS empty vector control; TRV-*HaXTH2*, transient *HaXTH2* silencing line; OE-EV, overexpression empty vector control; OE-*HaXTH2*, transient *HaXTH2* overexpression line. Black arrows mark haustorial attachment sites. Scale bar = 5 mm. (**B**) Transverse histological sections of sunflower roots at haustorial attachment sites. Oc, *O. cumana*; Vs, sunflower root vascular tissue. Red arrows indicate xylem bridges formed to establish vascular connections between parasite and host roots. Scale bar = 100 μm. Data are representative of three independent experiments. (**C**) Quantification of germinated *O. cumana* seeds in all treatment and control groups at 15 dpi under hydroponic culture. Data are means ± SD from three independent experiments, each with 18 biological replicates per group. Asterisks indicate statistically significant differences between experimental and control groups. (**D**) Quantification of parasitic tubercle numbers in each group at 15 dpi under hydroponic inoculation. Data are means ± SD from three independent experiments, each with 18 biological replicates per group. Asterisks indicate statistically significant differences between experimental and control groups. Asterisks indicate statistically significant differences between experimental and control groups (one-way ANOVA with Dunnett’s post-hoc test; *** p < 0.001; ns, not significant).

First, the number of germinated *O. cumana* seeds attached to sunflower roots was quantified at 15 dpi. No significant differences in seed germination counts were detected among *HaXTH2* overexpression, *HaXTH2* virus-induced gene silencing (VIGS), and empty vector control groups, with approximately 43 germinated seeds recorded per treatment (Figure 2C). This result indicates that *HaXTH2* does not modulate *O. cumana* seed germination and that it functions exclusively at post-germination stages, including haustorial penetration and subsequent parasitic development.

Stereomicroscopic observation of haustorium and tubercle development revealed distinct phenotypic gradients across genotypes. The VIGS-silenced line TRV-*HaXTH2* only formed rudimentary haustoria; compared with the Tobacco rattle virus (TRV) empty vector control (TRV-EV), haustorial growth was markedly retarded, and no mature swollen parasitic tubercles were produced. Roots of the overexpression empty vector line (OE-EV) merely generated tiny haustoria without obvious tubercle expansion. In contrast, haustoria formed on OE-*HaXTH2* overexpressing roots elongated normally and developed fully mature parasitic tubercles (Figure 2A).

To dissect the cytological mechanism by which *HaXTH2* mediates parasitism at the cellular level, paraffin sections were prepared from the haustorium-host root junction of all treatment groups and subjected to Safranin O-Fast Green double staining to visualize cell wall structural variations. The staining specificity of the two dyes is described as follows: Safranin O is a cationic basic dye that specifically binds to phenolic hydroxyl groups in lignin and ester bonds in suberin, staining lignified thickened secondary cell walls and xylem bridges bright red. Fast Green is an anionic counterstain that electrostatically associates with non-lignified primary cell walls and cytoplasm, labeling parenchymal tissues light green.

Histological section observations demonstrated that haustoria from the TRV-EV control successfully penetrated the host cortex and established vascular connections with root vascular bundles, generating a moderate number of xylem bridge cells. In contrast, the invasion sites of TRV-*HaXTH2* silenced lines exhibited drastically elevated lignification levels, accompanied by a sharp reduction in xylem bridge abundance. Meanwhile, the haustorium-host interface of OE-*HaXTH2* overexpressing lines was predominantly stained with Fast Green, suggesting loosened primary cell walls with minimal lignin deposition in this region (only weak lignified signals were detected in vascular tissues). Moreover, the number of xylem bridge cells formed upon *O. cumana* invasion was significantly higher than that in the OE-EV control, whereas fewer xylem bridge cells were observed in the OE-EV roots (Figure 2B). Quantitative analysis of parasitic tubercles at 15 days post inoculation demonstrated that TRV-HaXTH2 lines exhibited a significantly reduced number of parasitic tubercles, whereas OE-HaXTH2 lines showed a marked increase compared with their respective empty vector controls (Figure 2D).

Collectively, these cytological observations fully corroborate the macroscopic phenotypic data. Overexpression of *HaXTH2* loosens host primary cell walls and represses lignin-based defensive responses at early infection stages, which facilitates haustorial penetration through cortical tissues and robust xylem bridge formation, ultimately supporting the normal development of *O. cumana* parasitic tubercles.

### 2.3. Functional Analyses of XTH2 Homolog HaXTH2-1 and OcXTH2

**Figure 3:**
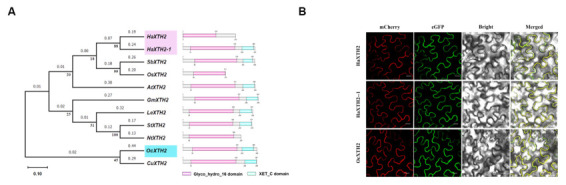
Phylogenetic relationships, domain architectures, and subcellular localization of XTH2 homologs from sunflower and *O. cumana*. (**A**) Phylogenetic analysis of HaXTH2, HaXTH2-1, OcXTH2, and their homologous proteins retrieved from 11 plant taxa, including *H. annuus*, *A. thaliana*, *O. cumana*, *S. lycopersicum*, *G. max*, *O. sativa*, *S. bicolor*, *C. australis*, *S. tuberosum*, and *N. benthamiana*. The phylogenetic tree was constructed based on the XTH2 protein sequences from these plant taxa. (**B**) Subcellular localization of HaXTH2, HaXTH2-1, and OcXTH2 fusion proteins. *Agrobacterium tumefaciens* GV3101 harboring pCambia2300-*HaXTH2*/*HaXTH2-1/OcXTH2*-eGFP constructs was infiltrated into *Nicotiana benthamiana* leaves. Fluorescence signals were observed using confocal microscopy at 48 h post-infiltration (hpi). mCherry served as a plasma membrane marker. Scale bars = 20 μm. Data are representative of three independent experiments.

Through NCBI database searching, we identified *HaXTH2-1* as the closest homolog of *HaXTH2* in sunflower. Genome-wide and transcriptome-wide screening of *O. cumana* revealed only one highly homologous gene, which was named *OcXTH2* (Figure S1). To investigate their phylogenetic relationships and domain architectures, we conducted domain prediction analyses and constructed a phylogenetic tree using XTH2 proteins from 11 plant species, including *Helianthus annuus*, *Arabidopsis*, *Orobanche cumana*, *Solanum lycopersicum*, *Glycine max*, *Oryza sativa*, *Sorghum bicolor*, *Cuscuta australis*, *Solanum tuberosum*, and *Nicotiana benthamiana*. Phylogenetic analysis revealed that HaXTH2, HaXTH2-1 were closely related, whereas sunflower and *O. cumana* XTH2s were evolutionarily distant. Domain prediction analysis indicated that all identified XTH2 proteins contained the conserved Glyco_hydro_16 and XET_C domains, except for HaXTH2, which harbored an incomplete XET_C domain (Figure 3A). To determine the subcellular localization of the three XTH2 proteins, GFP fusion constructs of HaXTH2, HaXTH2-1, and OcXTH2 were generated. Each construct was transiently co-expressed with the plasma membrane marker mCherry in *Nicotiana benthamiana* leaves via *Agrobacterium tumefaciens*-mediated infiltration. Confocal microscopy at 48 h post-infiltration revealed that all three XTH2-GFP fusion proteins localized to the cell wall (Figure 3B).

**Figure 4:**
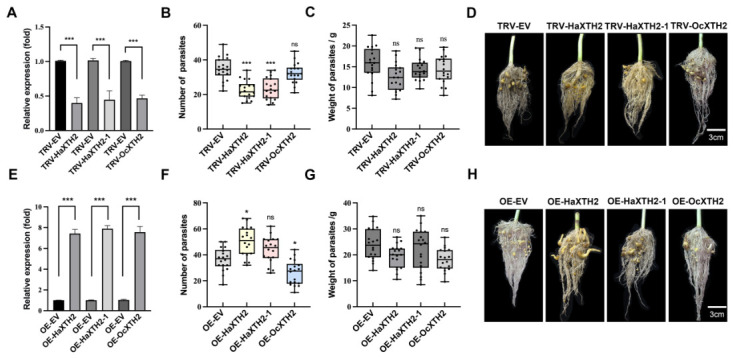
Functional characterization of *HaXTH2*, *HaXTH2-1*, and *OcXTH2* in regulating *O. cumana* parasitism. (**A**,**E**) Transcript levels of *HaXTH2*, *HaXTH2-1*, and *OcXTH2* in sunflower roots following transient silencing (**A**) or overexpression (**E**) at 45 days post-inoculation (dpi) with *O. cumana*. Data are means ± SD of three biological replicates (*n* = 3). (**B**,**F**) Quantification of *O. cumana* tubercles on roots of TRV-*HaXTH2*, TRV-*HaXTH2-1*, TRV-*OcXTH2* plants (**B**) and OE-*HaXTH2*, OE-*HaXTH2-1*, OE-*OcXTH2* plants (**F**) at 45 dpi. Data are means ± SD from three independent experiments, each with 18 biological replicates per group. Asterisks indicate statistically significant differences between experimental and control groups. (**C**,**G**) Fresh weight of roots before and after *O. cumana* inoculation in silenced (**C**) and overexpressing (**G**) plants. Data are means ± SD from three independent experiments, each with 18 biological replicates per group. No significant differences were observed between any groups (ns, not significant). (**D**,**H**) Representative phenotypes of *O. cumana* parasitism on roots of TRV-*HaXTH2*, TRV-*HaXTH2-1*, TRV-*OcXTH2* plants (**D**) and OE-*HaXTH2*, OE-*HaXTH2-1*, OE-*OcXTH2* plants (**H**) at 45 dpi. TRV-EV and OE-EV represent the empty vector controls for the TRV and OE groups, respectively. Scale bars = 3 cm. Asterisks indicate statistically significant differences between experimental and control groups (one-way ANOVA with Dunnett’s post-hoc test; * p < 0.05, *** p < 0.001; ns, not significant).

To investigate the roles of *XTH2s* during *O. cumana* parasitism, we employed virus-induced gene silencing (VIGS)-based knockdown and transient overexpression of *HaXTH2*, *HaXTH2-1*, and *OcXTH2* in the sunflower cultivar HN3638 using an *Agrobacterium*-mediated seed soaking assay (SSA) [38]. RT-qPCR analysis confirmed effective suppression of transcript levels in TRV-*HaXTH2*, TRV-*HaXTH2-1*, and TRV-*OcXTH2* plants, with gene expression levels significantly reduced by approximately 62%, 50%, and 55%, respectively, compared to the TRV-EV control group (Figure 4A). At 45 days post-inoculation (dpi) with *O. cumana*, we conducted a statistical analysis of parasitic infections on sunflower roots. Compared with the control group, the pTRV2-*HaXTH2* plants exhibited a reduction in both the number of tubercles and fresh weight of *O. cumana* attached to sunflower roots. The pTRV2-*HaXTH2-1* group showed a significant decrease in tubercle number, accompanied by a decrease in parasitic biomass. In contrast, no significant changes in tubercle number or fresh weight were observed in the pTRV2-*OcXTH2* group compared to the control (Figure 4B–D). We next examined the effects of *XTH2* overexpression on *O. cumana* parasitism. RT-qPCR analysis confirmed that transcript levels of *XTH2* were significantly elevated in OE-*HaXTH2*, OE-*HaXTH2-1*, and OE-*OcXTH2* sunflower roots, showing 7.4-fold, 7.9-fold, and 7.7-fold increases, respectively, compared to the empty vector control (Figure 4E). At 45 days post-inoculation with *O. cumana*, OE-*HaXTH2* and OE-*HaXTH2-1* plants exhibited significantly higher numbers of parasitic tubercles compared to the control group. In contrast, overexpression of *OcXTH2* led to a significant reduction in both the number and biomass of *O. cumana* tubercles (Figure 4F–H). Collectively, these results demonstrate that *HaXTH2* and *HaXTH2-1* enhance *O. cumana* parasitism, whereas *OcXTH2* suppresses parasitism by reducing both tubercle number and parasitic biomass.

### 2.4. Identification of Functional Domains in XTH2 Homologs

**Figure 5:**
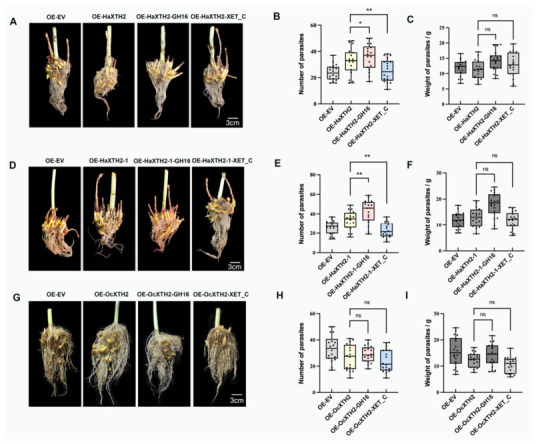
The GH16 domain is essential for *HaXTH2* and *HaXTH2-1* to promote *O. cumana* parasitism. (**A**,**D**,**G**) Representative phenotypes of *O. cumana* parasitism on sunflower roots overexpressing the full-length or domain-truncated constructs of *HaXTH2* (**A**), *HaXTH2-1* (**D**), and *OcXTH2* (**G**) at 45 dpi. OE-EV indicates the empty vector control. Scale bars = 3 cm. (**B**,**E**,**H**) Quantification of *O. cumana* tubercles on roots overexpressing the full-length or domain-truncated constructs of *HaXTH2* (**B**), *HaXTH2-1* (**E**), and *OcXTH2* (**H**) at 45 dpi. Data are means ± SD from three independent experiments, each with 18 biological replicates per group. Asterisks indicate statistically significant differences between experimental and control groups (one-way ANOVA with Dunnett’s post-hoc test; * p < 0.05, ** p < 0.01; ns, not significant). (**C**,**F**,**I**) Fresh weight of roots overexpressing the full-length or domain-truncated constructs before and after *O. cumana* inoculation. Data are means ± SD from three independent experiments, each with 18 biological replicates per group. No significant differences were observed between any groups (ns, not significant).

To identify the minimal functional units of these proteins during *O. cumana* parasitism, we generated truncated constructs based on the domain architecture of XTH proteins (Figure S2). Specifically, the GH16 and XET_C domains were separately cloned into the pCambia-2300 overexpression vector and transiently overexpressed in the sunflower cultivar HN3638 via *Agrobacterium*-mediated transformation. At 45 dpi with *O. cumana*, tubercle number was quantified, and root biomass was measured. Notably, overexpression of the *HaXTH2*-GH16 domain alone resulted in a significant increase in *O. cumana* tubercle number compared to overexpression of full-length *HaXTH2*, whereas overexpression of the *HaXTH2*-XET_C domain led to a significant reduction in tubercle number relative to full-length *HaXTH2* overexpression (Figure 5A–C). Similarly, overexpression of the *HaXTH2-1*-GH16 domain alone resulted in a further increase in *O. cumana* tubercle number compared to overexpression of full-length *HaXTH2-1*. Meanwhile, overexpression of the *HaXTH2-1*-XET_C domain led to a significant reduction in tubercle number relative to the full-length overexpression control (Figure 5D,E), although no consistent pattern was observed in parasitic biomass (Figure 5F). In contrast, neither overexpression of *OcXTH2*-GH16 nor *OcXTH2*-XET_C significantly affected *O. cumana* tubercle number or parasitic biomass compared to overexpression of full-length *OcXTH2* (Figure 5G–I).

Taken together, these results suggest that during *O. cumana* parasitism, the GH16 domains of *HaXTH2* and *HaXTH2-1* may facilitate parasitism by degrading xyloglucan and contributing to cell wall loosening or remodeling. These findings indicate that the GH16 domains of *HaXTH2* and *HaXTH2*-1 are crucial domains influencing *O. cumana* parasitism.

### 2.5. Identification of Critical Residues Governing XTH2 Function

**Figure 6:**
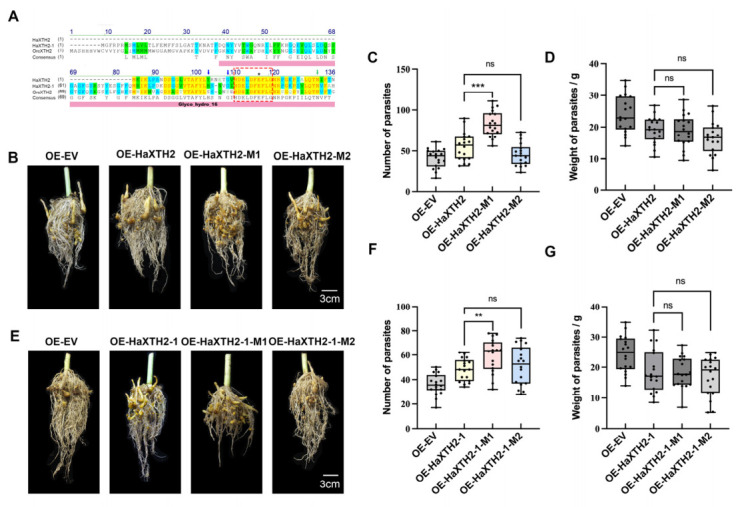
Identification of key amino acid residues in HaXTH2 and HaXTH2-1 governing *O. cumana* parasitism. (**A**) Amino acid sequence alignment of *HaXTH2* homologs and *OcXTH2*. Blue arrows indicate the divergent residues between sunflower and *O. cumana* sequences. (**B**,**E**) Representative phenotypes of *O. cumana* parasitism on sunflower roots at 45 days post-inoculation (dpi) after overexpression of *HaXTH2* (**B**), *HaXTH2-1* (**E**), and their site-directed mutants. OE-EV indicates the empty vector control. Scale bars = 3 cm. (**C**,**F**) Quantification of *O. cumana* tubercles on roots overexpressing the full-length or mutant constructs of *HaXTH2* (**C**) and *HaXTH2-1* (**F**) at 45 dpi. Data are means ± SD from three independent experiments, each with 18 biological replicates per group. Asterisks indicate statistically significant differences between experimental and control groups. (**D,G**) Fresh weight of roots overexpressing the full-length or mutant constructs before and after *O. cumana* inoculation for *HaXTH2* (**D**) and *HaXTH2-1* (**G**). Data are means ± SD from three independent experiments, each with 18 biological replicates per group. No significant differences were observed between any groups (ns, not significant). Asterisks indicate statistically significant differences between experimental and control groups (one-way ANOVA with Dunnett’s post-hoc test; ** p < 0.01, *** p < 0.001; ns, not significant).

To accurately identify the essential active sites influencing *O. cumana* parasitism, the amino acid sequences of the GH16 region of the XTH2 proteins from sunflower and the parasitic plant *Cuscuta* were aligned. Sequence alignment revealed that all three XTH2 sequences contain a conserved characteristic motif, DEIDFEFLG (highlighted by red dashed boxes in Figure S1), which is essential for XET/XTH enzymatic activity. Additionally, these sequences also possess a critical first glutamate residue (E) (marked by black asterisks) and an asparagine residue (N) at the N-glycosylation site (marked by green arrows). Analysis of the conserved domains of HaXTH2, HaXTH2-1, and OcXTH2 proteins revealed that the serine residue (S104) in OcXTH2 is replaced by a tyrosine residue (Y20) in HaXTH2 and by a histidine residue (H96) in HaXTH2-1. Additionally, the serine residue (S108) in OcXTH2 is substituted by a glycine residue (G25) in HaXTH2 and by a glycine residue (G100) in HaXTH2-1 (both sites are marked with blue arrows). Notably, additional amino acid differences between *HaXTH2*/*HaXTH2-1* and *OcXTH2* were identified upstream of the conserved motif. Based on these observations, we hypothesized that Tyr20 and Gly25 in *HaXTH2*, as well as His96 and Gly100 in *HaXTH2-1*, play critical roles during *O. cumana* parasitism. To test this hypothesis, we performed site-directed mutagenesis to generate overexpression constructs carrying specific amino acid substitutions, which were then transiently overexpressed in sunflower roots, followed by *O. cumana* inoculation to assess parasitic phenotypes. Notably, when Tyr20 in *HaXTH2* was mutated to serine (hereafter referred to as the *HaXTH2*-M1 mutant), we observed a significant increase in *O. cumana* tubercle number compared to plants overexpressing wild-type *HaXTH2* (Figure 6A,B), with no change in root biomass (Figure 6C). Furthermore, we generated a double mutant by introducing a G25S substitution into the *HaXTH2*-M1 background (hereafter referred to as the *HaXTH2*-M2 mutant). However, compared to plants overexpressing wild-type *HaXTH2*, the *HaXTH2*-M2 mutant did not result in significant changes in *O. cumana* tubercle number (Figure 5A,B), nor were significant differences observed in root biomass (Figure 6C). Based on the *HaXTH2*-M1 background, a second mutant carrying the G25S substitution (*HaXTH2*-M2) was generated. However, compared with plants overexpressing *HaXTH2*, the M2 mutation did not lead to a significant change in the number of *Orobanche* parasites (Figure 6A,B), nor did it significantly affect biomass (Figure 6C). Similarly, for *HaXTH2-1*, the H96 residue was mutated to serine to generate *HaXTH2-1*-M1. An overexpression vector was constructed and transiently expressed in sunflowers, and the plants were inoculated with *O. cumana* to assess the parasitic phenotype. Compared with *HaXTH2-1*-overexpressing controls, *HaXTH2-1*-M1 plants exhibited a significant increase in the number of *O. cumana* attachments (Figure 6D,E), whereas the biomass remained unchanged (Figure 6F). On the *HaXTH2-1*-M1 background, a second mutant with the G100S substitution (*HaXTH2-1*-M2) was generated. However, no significant difference in parasite number (Figure 6D,E) or biomass (Figure 6F) was observed between *HaXTH2-1*-M2 and control plants overexpressing *HaXTH2-1*. We predicted HaXTH2/HaXTH2-1 structures with AlphaFold2 and visualized them in PyMOL 3.1 (Figure S3). Y20S and H96S do not disrupt overall GH16 folding but destroy substrate-binding and catalytic side chains. Catalytically inactive mutants act as dominant-negative competitors, disrupting endogenous cell wall remodeling and elevating *O. cumana* colonization. Collectively, these mutagenesis results indicate that residue Y20 in *HaXTH2* and residue G100 in *HaXTH2-1* are key functional sites regulating *O. cumana* parasitism.

**Figure 7:**
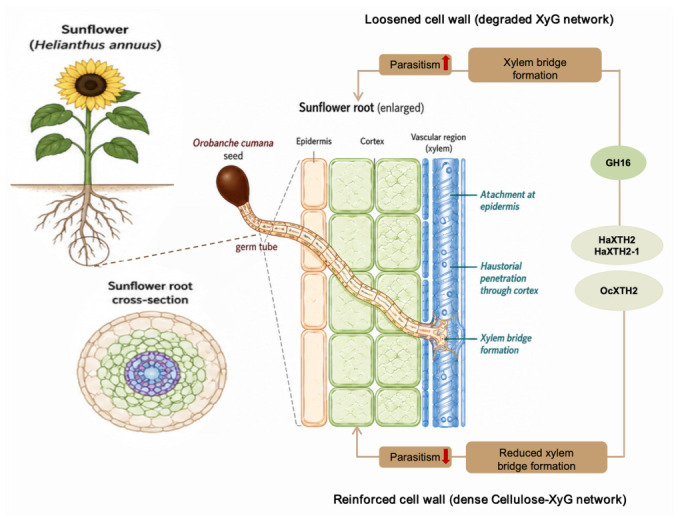
Working model illustrating the antagonistic regulatory functions of *HaXTH2*, *HaXTH2-1*, and *OcXTH2* during parasitic interaction between sunflower and *O. cumana*. Upon infection by germinated *O. cumana* seeds, host *HaXTH2* and its paralog *HaXTH2-1* are upregulated and localized to the cell walls of sunflower roots. These two proteins remodel xyloglucan (XyG) networks via the conserved GH16 (glycoside hydrolase family 16) domain, which loosens primary cell walls and represses lignin-based immune defenses. The weakened cell wall barrier facilitates penetration of parasite haustoria through cortical cells and the formation of xylem bridges, ultimately promoting parasitism. In contrast, parasite-derived *OcXTH2* accumulates at host cell walls and triggers cell wall reinforcement, accompanied by dense cellulose-XyG crosslinks, elevated lignin deposition, and callose accumulation. This strengthened physical barrier blocks haustorial invasion and impairs xylem bridge assembly, thereby suppressing parasitic colonization.

Based on all phenotypic, gene expression, subcellular localization, domain truncation and site-directed mutation data obtained in this work, we established a working model to illustrate the antagonistic regulatory roles of *XTH2* homologs during the parasitic interaction between sunflower and *O. cumana* (Figure 7).

## 3. Discussion

In this study, we elucidated the mechanism by which GH16 domain-containing *XTH2* genes regulate sunflower defense responses to *O. cumana* parasitism. Our results show that host *HaXTH2* is significantly induced during parasitic infection (Figure 1). Paraffin section staining revealed divergent cell wall structures and lignin deposition patterns modulated by host *HaXTH2* and parasitic *OcXTH2*, providing morphological evidence for their antagonistic functions in cell wall remodeling. However, histological staining only provides qualitative observations and cannot quantify in planta lignin accumulation or distinguish the catalytic differences between the two *XTH* homologs. Future work will combine recombinant protein biochemical assays and quantitative lignin detection to clarify the relationship between *XTH* enzyme activity, cell wall metabolism, and host anti-parasitic immunity. Overexpression assays confirmed that sunflower *HaXTH2* and *HaXTH2-1* promote *O. cumana* tubercle formation, whereas parasite-derived *OcXTH2* significantly suppresses parasitism (Figure 4). Consistent with previous reports in other parasitic systems, haustorium-expressed *CcXTH* facilitates host cell wall loosening and Cuscuta invasion [39,40], while tomato *LeXTH1* enhances cell wall compactness to resist parasitic infection [37]. The opposite effects of host and parasitic XTH2 homologs observed here suggest that *HaXTH2*/*HaXTH2-1* weaken sunflower cell wall rigidity to facilitate haustorial penetration, whereas *OcXTH2* restricts parasitism through cell wall stabilization. Such functional divergence may arise from distinct substrate preferences or catalytic properties, leading to differential outcomes in cell wall remodeling during host–parasite interaction. In addition to lignification and xyloglucan remodeling, callose deposition represents a critical cell wall defense against root parasitic weeds. Host plants rapidly activate callose synthases at haustorial penetration sites to form impermeable physical barriers that block nutrient delivery and restrict parasite expansion. Future studies will further explore the potential signaling crosstalk between XTH-mediated xyloglucan modification and callose-based defense during sunflower–*O. cumana* parasitism.

The contrasting functions of host *HaXTH2*/*HaXTH2-1* and parasitic *OcXTH2* in regulating *O. cumana* parasitism suggest that these homologs may exert distinct effects on host cell wall dynamics. Xyloglucan (XyG) fragments are known to act as damage-associated molecular patterns (DAMPs) that can trigger plant immune responses upon receptor recognition [41], and similar antagonistic *XTH* functions have been observed in other parasitic systems such as *Cuscuta* [39]. Host *HaXTH2*/*HaXTH2-1* preferentially degrade sunflower endogenous XyG to generate weakly immunogenic oligosaccharides, which fail to trigger robust lignin and callose-based defenses and thus facilitate haustorial penetration. By contrast, parasitic *OcXTH2* targets parasite-originated XyG at the host–parasite interface, releasing highly immunogenic DAMP signals to activate localized lignin deposition and callose crosslinking that restrict parasitic colonization. This substrate preference divergence accounts for their opposing regulatory roles in parasitism, though whether phenotypic differences stem from distinct XyG oligosaccharide profiles or DAMP potency awaits further verification. Future biochemical assays with recombinant XTH proteins and defense transcriptome profiling will dissect the precise biochemical basis of this functional divergence. Our current data solidly confirm that *HaXTH2*/*HaXTH2-1* promote *O. cumana* infection whereas *OcXTH2* inhibits it, with the GH16 domain and key residues Tyr20/His96 as indispensable functional determinants, while downstream immune cascades remain to be fully resolved. Accumulating studies have revealed the diverse functions of plant *XTH* family genes across multiple species. Apple *MdXTH*, which harbors conserved GH16 and XET domains, participates in fruit texture regulation and responds to diverse biotic and abiotic stresses as well as hormonal signals [42]. These findings demonstrate the functional and regulatory diversity of *XTH* members, which is further supported by our results. Domain analysis verified that *HaXTH2*, *HaXTH2-1*, and *OcXTH2* all contain complete GH16 and C-terminal XET domains (Figure 3A). Further truncation assays confirmed the GH16 domain serves as the core functional module governing sunflower–*O. cumana* interaction (Figure 5). XyG degradation products are well documented to modulate plant cell wall elasticity and rigidity [43,44]. The enhanced parasitism caused by GH16 domain overexpression indicates that this domain primarily functions as a hydrolase, modulating cell wall loosening to facilitate haustorium development and parasitic colonization.

In addition to functional versatility in developmental regulation, *XTH* genes also participate in abiotic stress adaptation via cell wall remodeling. For instance, multiple *MaXTH* members in banana differentially regulate cold tolerance, with distinct expression patterns between cold-tolerant and cold-sensitive cultivars [45]. Such evidence further confirms the essential role of *XTHs* in mediating xyloglucan rearrangement and cell wall dynamic remodeling [46,47,48]. Consistent with these findings, our study demonstrates that *HaXTH2*, *HaXTH2-1*, and *OcXTH2* modulate sunflower–*O. cumana* interactions by regulating xyloglucan incorporation and cell wall integrity. Host HaXTH2/HaXTH2-1 upregulation during infection promotes primary cell wall modification to facilitate parasite penetration, whereas *OcXTH2* impairs cell wall assembly to suppress parasitism. The peak XET activity detected at the haustorium-host interface during *Cuscuta* parasitism further implies stage-specific XTH functions during parasitic colonization, raising the possibility of divergent XET domain roles in haustorium formation and subsequent parasitic development. Post-translational modifications, especially ubiquitination, are core regulatory mechanisms governing plant biotic stress immunity, wherein E3 ubiquitin ligases modulate pathogen recognition and downstream immune signaling [49,50]. Our site-directed mutagenesis results further identified key functional residues determining *XTH2*-mediated parasitism regulation. Mutation of Tyr20 in *HaXTH2* and His96 in *HaXTH2-1* both significantly increased *O. cumana* colonization, verifying that these two amino acid sites are indispensable for the biological functions of *HaXTH2* and *HaXTH2-1* during sunflower–parasite interaction (Figure 6A–F).

Plants activate multiple defense strategies to resist adverse stresses, including antioxidative responses, cell wall reinforcement, and JA/SA-mediated hormone signaling, which collectively contribute to host defense against *O. cumana* parasitism [51,52]. Consistent with our findings, maximal XET activity is detected at the host-haustorium interface during parasitic infection, supporting the critical involvement of the XET-C domain of XTH2 in haustorial formation and parasitic progression [53]. The identification of these key XTH2 functional residues and domains provides valuable target genes for sunflower resistance breeding. Pyramiding favorable *XTH2* resistance alleles with superior agronomic genes facilitates the breeding of high-yield and parasite-resistant sunflower cultivars. In addition, genome editing of key functional residues and chemical modulation of XET activity combined with ABA treatment can serve as effective and eco-friendly strategies for field *O. cumana* control. Overall, this study expands our understanding of *XTH*-mediated cell wall regulatory mechanisms during host–parasite interactions and offers promising resources for crop stress improvement and yield stabilization. Future work will further explore the functional diversification of XTH domains and their application potential in molecular breeding.

## 4. Materials and Methods

### 4.1. Plant Materials and Growth Conditions

The sunflower variety Huannong HN3638 (HN3638) was used as the host plant in this study. The seeds of *O. cumana* were collected from Wuchuan County, Hohhot City (G minor), Inner Mongolia Autonomous Region. For the greenhouse soil cultivation system used for *O. cumana* inoculation, a nutrient substrate was prepared by mixing seedling substrate and vermiculite at a 1:1 volume ratio. Approximately 45 g of the nutrient substrate was thoroughly mixed with 0.018 g of *O. cumana* seeds, and then one sunflower seed was sown at a depth of about 2–3 cm in a nutritive bowl (15 cm in height, 10 cm in diameter). The cultivation setup was maintained in a controlled climate chamber for 50 days under a photoperiod/temperature cycle of 12 h light at 25 °C (±2 °C) and 12 h dark at 22 °C (±2 °C). Once *O. cumana* seedlings emerged, sunflower roots were carefully washed with sterile water, and the *O. cumana* inoculation phenotypes were recorded and sampled immediately. All collected samples were stored at −80 °C for subsequent experimental analysis. Each treatment group contained 18 biological replicates to ensure statistical reliability. On the 25th day after sowing, sunflower plants were gently removed from the seedling pots, their roots were rinsed with clean water to remove residual substrate, and the number of parasitic nodules and the overall parasitic phenotypes were observed and documented in detail.

### 4.2. Vector Construction and SSA Transient Transformation

Target sequences of 300 bp were selected for *HaXTH2*, *HaXTH2-1*, and *OcXTH2*, ensuring less than 20 nt similarity with other sunflower and *O. cumana* genes. The target gene interference fragments were cloned into the pTRV2 vector and co-transformed with the sunflower gene pTRV2, then co-transformed with pTRV1 into *Agrobacterium* GV3101. For overexpression vectors, the PCR-amplified coding sequences (CDS) of the target genes were cloned into the pCambia2300-GFP vector and subsequently transformed into *Agrobacterium tumefaciens* GV3101. The *Agrobacterium* suspension for infiltration was then prepared.

SSA infection solution was prepared with sterile water, 10 mM 2-(N-morpholino) ethanesulfonic acid (MES), 10 mM MgCl_2_, 200-μm acetosyringone (AS), 5% prepared at a 1/100 ratio as an infection base solution. *Agrobacterium* cultures of pTRV1 and recombinant pTRV2 vectors were grown to optical density at 600 nm (OD_600_) = 1.0–1.5 and resuspended in the infection solution, mixed at a 1:1 ratio, and prepared as infection liquid for SSA. Sunflower seeds were peeled of inner and outer seed coatings, gently scratched with tweezers, and soaked in the infection liquid in darkness for 5 h. Seeds were placed in seedling pots with moist filter paper, cultured in a 25 °C light incubator for 5 days, then transferred to pots with 0.06 g of *O. cumana* seeds in the soil for 50 days. After 50 days, complete sunflower roots, including inoculated *O. cumana*, were extracted for qPCR testing, phenotypic observation, and biomass statistics. All biomass data were measured as fresh weight.

For parasitic biomass measurement, intact sunflower root systems with attached *O. cumana* tubercles were harvested at 45 days post-inoculation. The roots were gently rinsed to remove soil particles, surface water was blotted dry with filter paper, and the combined fresh weight of host roots and parasitic tubercles was determined using an electronic analytical balance [38].

### 4.3. Subcellular Localization Analysis

For transient subcellular localization assays, two independent systems were applied: *N. benthamiana* leaf agroinfiltration and onion epidermal cell transformation. For GFP fusion vector construction, the full-length coding sequences of *HaXTH2*, *HaXTH2-1* and *OcXTH2* without stop codons were amplified by specific primers, then inserted into the pCambia2300-GFP vector to generate 35S::target gene-GFP C-terminal fusion constructs. For plasma membrane co-localization verification, the mCherry plasma membrane marker vector 35S::PIP2A-mCherry was co-transformed with each GFP fusion construct at an equal Agrobacterium OD ratio.

*Agrobacterium tumefaciens* GV3101 strains harboring individual GFP fusion plasmids and the mCherry marker plasmid were cultured, resuspended to OD_600_ = 1.0, and mixed equally before infiltration into 4-week-old *N. benthamiana* leaves. Fluorescence signals were detected via confocal microscopy at 48 h post-infiltration, with mCherry signals serving as plasma membrane reference.

For the onion epidermal cell assay, the same recombinant pCambia2300-GFP fusion constructs were transformed into GV3101. Healthy onion bulbs were surface-sterilized, and the inner epidermal layers (3rd–4th scales) were peeled into approximately 0.5 cm × 0.5 cm segments. The epidermal strips were immersed in the Agrobacterium suspension for 30 min, blotted dry on sterile filter paper, and then placed on Murashige and Skoog (MS) medium for co-cultivation in the dark at 25 °C for 16–24 h. Distilled water was used as a mock control. For plasmolysis induction, the epidermal strips were mounted in 100 μL of sucrose solution at the indicated concentrations on glass slides, covered with coverslips, and incubated at room temperature (25 °C) for 15 min. GFP fluorescence signals were observed using a confocal laser scanning microscope.

### 4.4. RNA Extraction, PCR, RT-qPCR, and Subcellular Localization Analysis

Total RNA was extracted using Trizol reagent (TransGen Biotech Co., Ltd., Beijing, China) according to the manufacturer’s protocol. Potential genomic DNA contamination was eliminated by DNase I treatment. RNA quality and concentration were assessed with a NanoDrop2000 spectrophotometer (Thermo Fisher Scientific, Waltham, MA, USA). First-strand cDNA was synthesized from equal amounts of total RNA using the HiScript III RT SuperMix for qPCR kit (Vazyme Biotech Co., Ltd., Nanjing, China). For qPCR analysis, specific primers were designed, and HaActin (Accession No. AF282624) and OcActin (Unigene ID: Cluster-9748.42356) were used as internal reference genes for sunflower and *O. cumana*, respectively. Quantitative PCR was performed on a qTOWER2.2 real-time PCR system (Analytik Jena GmbH, Jena, Germany) using TransStart Tip Green qPCR SuperMix (TransGen Biotech Co., Ltd., Beijing, China). The amplification program was 95 °C for 2 min, followed by 40 cycles of 95 °C for 15 s and 60 °C for 30 s, with a melting curve analysis to verify product specificity. Relative expression levels were calculated using the 2^−ΔΔCt^ method. Each reaction was run with three technical replicates, and the data were obtained from three independent biological replicates. Statistical analysis was conducted with three biological replicates.

We further quantified the transcript abundance of all full-length and domain-truncated XTH2 constructs via the above RT-qPCR system to normalize overexpression levels. All target fragments were driven by the identical CaMV 35S promoter within the pCambia2300 backbone. Only sunflower seedlings with comparable relative expression of exogenous transgenes (no statistically significant differences across groups) were selected for subsequent *O. cumana* parasitism phenotypic assays, to exclude phenotypic bias caused by variable transgene expression.

### 4.5. Hairy Root Transformation of Sunflower and Orobanche cumana Inoculation System

The *Rhizobium rhizogenes* strain Ar.Qual was used to deliver overexpression vectors carrying target genes into sunflower to induce hairy root formation via genetic transformation. The transformation procedure was performed as described below. Briefly, 2 μL of plasmid DNA was mixed with 50 μL Ar.Qual competent cells, incubated on ice for 5 min, flash-frozen in liquid nitrogen for 5 min, and immediately heat-shocked in a 37 °C water bath for 5 min. After a further 5 min of incubation on ice, 600 μL liquid LB medium was added to the mixture. The bacterial suspension was incubated at 28 °C with shaking at 200 rpm for 4 h, then spread onto solid LB medium supplemented with streptomycin and kanamycin for positive selection. Sunflower seedlings at 5–7 days after germination were cut at the stem approximately 2 cm above the cotyledonary node, and hypocotyl segments 1 cm in length were collected from the cut site. The hypocotyl explants were mechanically wounded by pricking 7–8 sites with a sterile needle, then immersed in Ar.Qual bacterial suspension (OD_600_ = 0.8, preparation detailed in Section 4.3) for 4 h at room temperature with gentle shaking at 75 rpm. Inoculated explants were transferred to a breeding tray lined with moist filter paper and incubated at 25 °C under light conditions for 7 days to induce hairy root generation.

For establishment of the hairy root hydroponic culture system: 12 cm square culture dishes were used, with a layer of rockwool laid at the bottom for support and water retention, covered by two layers of autoclaved qualitative filter paper moistened with sterile ddH_2_O. Sunflower seedlings with induced hairy roots were transplanted onto the filter paper and acclimated for 5 days at 25 °C under a 16 h light photoperiod; filter paper was maintained moist without standing water during acclimation. After pre-cultivation, 0.02 g of *O. cumana* seeds were wrapped in qualitative filter paper for surface sterilization. Seeds were sequentially treated with 2% sodium hypochlorite solution and 75% ethanol for 10 min each, thoroughly rinsed with sterile ddH_2_O, air-dried in a laminar flow hood, and evenly distributed around the root zone of sunflower seedlings. At 24 h post-inoculation, 1 mL of 100 nM rac-GR24 solution was applied to the roots of each seedling to stimulate *O. cumana* seed germination. Seedlings were cultured continuously under the same temperature and light regimes. Fifteen days after inoculation, parasitic phenotypes of *O. cumana* were observed and sampled under a stereomicroscope (Nikon SMZ18, Tokyo, Japan). Harvested root tissues were fixed in Formalin-Aceto-Alcohol (FAA) fixative (50% ethanol, 5% glacial acetic acid, and 3.7% formaldehyde) for subsequent paraffin section preparation.

### 4.6. Paraffin Section Preparation and Safranin O-Fast Green Double Staining

Fresh sunflower root tissues harvested from hydroponic culture were fixed in FAA solution for 16–24 h. Fixed samples were transferred to embedding cassettes lined with gauze and thoroughly rinsed with running tap water to remove residual FAA fixative. Subsequent serial ethanol dehydration was performed as follows: 30%, 50%, 70%, 85% and 95% ethanol, each for 60 min, followed by two rounds of absolute ethanol dehydration, 60 min per round. For tissue clearing: samples were incubated in 50% xylene for 60 min, then treated twice with pure xylene, 60 min each time. Cleared tissues were immersed in molten pure paraffin wax at 58 °C overnight [54].

Specimens were three-dimensionally oriented and embedded using a Leica EG1150H paraffin embedding system (Leica Microsystems GmbH, Wetzlar, Germany) according to anatomical orientation. After embedding, serial sections of 10 μm thickness were cut with a Leica RM2245 rotary microtome (Leica Microsystems GmbH, Wetzlar, Germany). Sections were floated on distilled water at 42 °C using a slide warmer, mounted onto glass slides, and dried at 42 °C for 48 h. Dried sections were rehydrated via a graded ethanol series: two incubations in pure xylene (10 min each), two incubations in absolute ethanol (10 min each), two incubations in 95% ethanol (5 min each), one incubation in 75% ethanol (5 min), one incubation in 50% ethanol (5 min), and a final brief rinse in pure distilled water.

For double staining: rehydrated sections were stained with 0.1% Safranin O solution (Sigma-Aldrich, St. Louis, MO, USA, Cat. No. S8884) in the dark for 90 min, followed by counterstaining with 0.2% Fast Green solution for 15 min. Stained sections were mounted with Entellan neutral mounting medium (Merck KGaA, Darmstadt, Germany) without air bubbles, and observed under a Nikon Eclipse Ts2R inverted microscope.

### 4.7. Phylogenetic Tree Analysis

To understand the correlation of protein domains between sunflower HaXTH2, HaXTH2-1, and OcXTH2, HMMER (https://www.ebi.ac.uk/Tools/hmmer/search/hmmscan, accessed on 8 June 2026) was utilized online for domain prediction and to construct a phylogenetic tree.

### 4.8. Site-Directed Mutagenesis

Site-directed mutagenesis of *HaXTH2* and *HaXTH2-1* was performed using the Mut Express^®^ II Fast Mutagenesis Kit V2 (Vazyme Biotech Co., Ltd., Nanjing, China, Cat. No. C214) according to the manufacturer’s instructions. Mutagenic primers were designed using CE Design V1.0 software. The target fragments were amplified by PCR with Phanta Max Super-Fidelity DNA Polymerase. PCR products were digested with Dpn I to eliminate the methylated template plasmids, followed by recombination using exonuclease II. The recombinant products were transformed into *Escherichia coli* DH5α competent cells. Positive clones carrying the desired mutations were verified by Sanger sequencing. All primers applied for vector construction described in the Methods section are listed in Table S1.

### 4.9. Statistical Analysis

Data were analyzed using SPSS software (version 25.0, IBM Corp., Armonk, NY, USA). Normality and homogeneity of variances were verified using Shapiro–Wilk and Levene’s tests, respectively. For data meeting parametric assumptions, one-way ANOVA followed by Dunnett’s post-hoc test was used for multiple comparisons against a single empty-vector control group, and Student’s *t*-test was used for two-group pairwise comparisons. Significance levels are indicated as * *p* < 0.05, ** *p* < 0.01, and *** *p* < 0.001; ns, not significant. All data are presented as means ± SD from at least three independent biological replicates.

## 5. Conclusions

In conclusion, this study reveals the divergent regulatory mechanisms of XTH family proteins during the parasitic interaction between sunflower and *O. cumana.* Host-derived *HaXTH2* and its paralog *HaXTH2-1* act as positive regulators of parasitism by mediating host cell wall remodeling. Specifically, overexpression of *HaXTH2* loosens host primary cell walls and represses lignin-based defense responses at the early infection stage, thereby facilitating haustorial penetration across cortical tissues and robust xylem bridge formation to support the full establishment of *O. cumana*. In contrast, the parasite-originated *OcXTH2* inhibits parasitic colonization by counteracting such cell wall modification processes. Functional domain assays further verified that the conserved GH16 domains of *HaXTH2* and *HaXTH2-1* possess xyloglucan endohydrolase (XEH) activity and constitute the core functional modules responsible for promoting parasitism. Additionally, site-directed mutagenesis pinpointed Tyr20 of *HaXTH2* and His96 of *HaXTH2-1* as vital amino acid residues that control sunflower susceptibility to *O. cumana* infection. Collectively, XTH homologs originating from both host sunflower and parasitic *O. cumana* exert opposing regulatory effects on host primary cell wall remodeling, and their combined enzymatic activities collectively shape cell wall modification status to determine the success of parasitic colonization and the establishment of sunflower–*O. cumana* interaction.

## Data Availability

The original contributions presented in this study are included in the article/Supplementary Materials. Further inquiries can be directed to the corresponding author.

## Supplementary Materials

The following supporting information can be downloaded at: https://www.mdpi.com/article/10.3390/plants15142222/s1, Figure S1. Amino acid sequence alignment of HaXTH2 homologs and OcXTH2. Figure S2. Schematic representation of domain truncations in HaXTH2, HaXTH2-1, and OcXTH2 proteins. Figure S3. AlphaFold2-predicted full-length 3D structures of wild-type and mutant HaXTH2 and HaXTH2-1 visualized in PyMOL 3.1. Table S1. Gene primer information.
