# Supplementary material for: Functional Analysis of the GH16 Domain-Containing XTH2 Homologs in Mediating Sunflower Response to Orobanche cumana Parasitism
Source: Plants (Basel). 2026 Jul 21;15(14):2222. doi: 10.3390/plants15142222 (PMC13417130; doi:10.3390/plants15142222)
Supplement: Supplementary file 1 [file plants-15-02222-s001.zip › 2026-7-14修 2-附图.pdf]

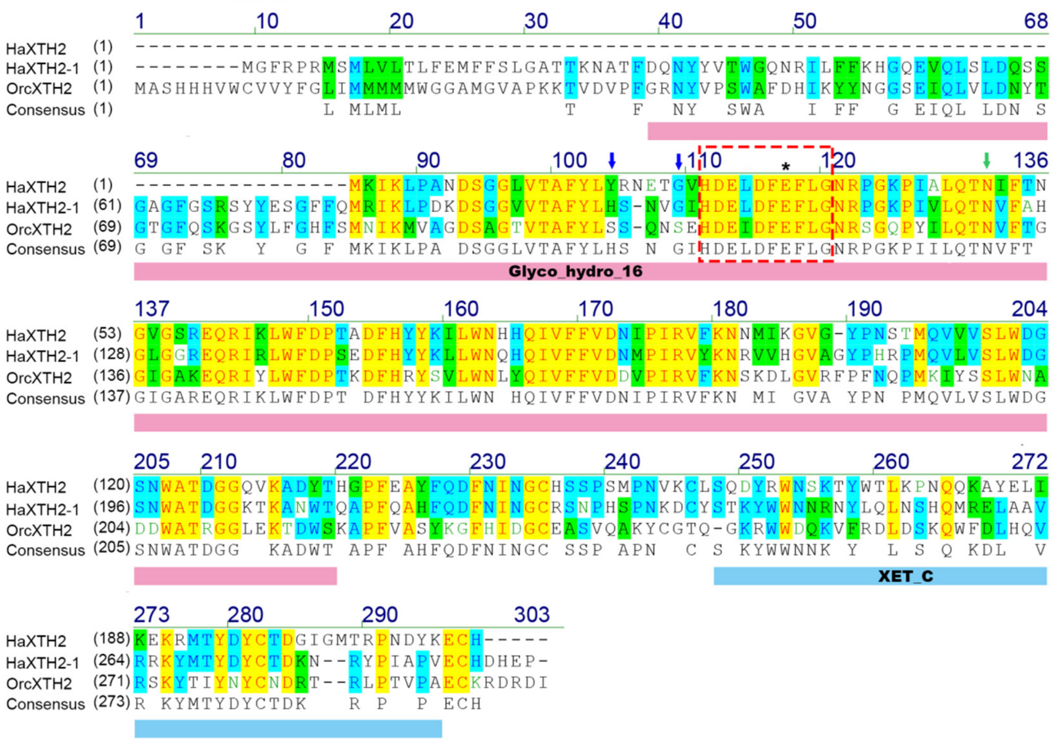

Figure S1. Amino acid sequence alignment of *HaXTH2* homologs and *OcXTH2*. The conserved motif involved in catalytic activity is indicated by the red dashed box. The glutamate residue (E) serving as the active site is marked with a black asterisk. Divergent amino acid residues between sunflower and *O. cumana* are indicated by blue arrows. Conserved N-glycosylation sites are marked with green arrows. The Glyco_hydro_16 and XET_C domains are highlighted in pink and blue, respectively.

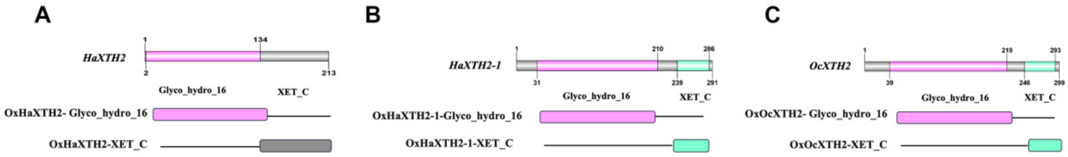

Figure S2. Schematic representation of domain truncations in HaXTH2, HaXTH2-1, and OcXTH2 proteins. Domain architecture analysis revealed that all three proteins contain both the Glyco_hydro_16 and XET_C domains, with the XET_C domain being incomplete in HaXTH2. The truncated regions corresponding to the full-length Glyco_hydro_16 domain are highlighted in pink across all three proteins. The full-length XET_C domains in HaXTH2-1 and OcXTH2 were truncated and are indicated in green. For HaXTH2, the truncated region, which encompasses the entire incomplete XET_C domain, is shown in gray.

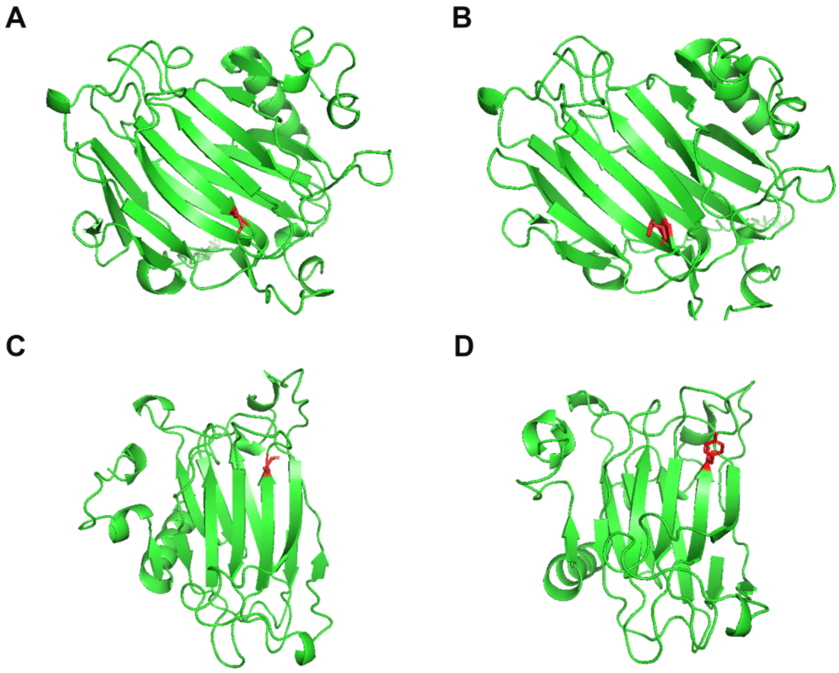

Figure S3. Full-length three-dimensional structural models of wild-type and mutant HaXTH2 and HaXTH2-1 predicted by AlphaFold2 and visualized via PyMOL 3.1. Green ribbons represent the full polypeptide chain of each protein, with the parallel β-sheet region corresponding to the conserved GH16 catalytic domain. Red sticks show the side chain of residue 20 or 96. (A) Wild-type HaXTH2 (Tyr20); (B) Y20S mutant HaXTH2; (C) Wild-type HaXTH2-1 (His96); (D) H96S mutant HaXTH2-1.
